# PpEst is a novel PBAT degrading polyesterase identified by proteomic screening of ***Pseudomonas pseudoalcaligenes***

**DOI:** 10.1007/s00253-016-7992-8

**Published:** 2016-11-21

**Authors:** Paal W. Wallace, Karolina Haernvall, Doris Ribitsch, Sabine Zitzenbacher, Matthias Schittmayer, Georg Steinkellner, Karl Gruber, Georg M. Guebitz, Ruth Birner-Gruenberger

**Affiliations:** 1 Research Unit for Functional Proteomics and Metabolic Pathways, Institute of Pathology, Medical University of Graz, Stiftingtalstrasse 24, 8010 Graz, Austria; 2 Austrian Centre of Industrial Biotechnology, Petersgasse 14, 8010 Graz, Austria; 3 Omics Center Graz, BioTechMed-Graz, Graz, Austria; 4 Austrian Centre of Industrial Biotechnology, Konrad Lorenz Strasse 20, 3430 Tulln, Austria; 5 Institute of Environmental Biotechnology, University of Natural Resources and Life Sciences, Konrad Lorenz Strasse 20, 3430 Tulln, Vienna Austria; 6 Institute of Molecular Biosciences, University of Graz, Humboldtstraße 50/III, 8010 Graz, Austria

**Keywords:** Proteomics, Poly(1,4-butylene adipate-co-terephthalate) (PBAT), Secretome, Polymer degradation, Polyesterase, Arylesterase

## Abstract

A novel esterase, PpEst, that hydrolyses the co-aromatic-aliphatic polyester poly(1,4-butylene adipate-co-terephthalate) (PBAT) was identified by proteomic screening of the *Pseudomonas pseudoalcaligenes* secretome. PpEst was induced by the presence of PBAT in the growth media and had predicted arylesterase (EC 3.1.1.2) activity. PpEst showed polyesterase activity on both whole and milled PBAT film releasing terephthalic acid and 4-(4-hydroxybutoxycarbonyl)benzoic acid while end product inhibition by 4-(4-hydroxybutoxycarbonyl)benzoic acid was observed. Modelling of an aromatic polyester mimicking oligomer into the PpEst active site indicated that the binding pocket could be big enough to accommodate large polymers. This is the first report of a PBAT degrading enzyme being identified by proteomic screening and shows that this approach can contribute to the discovery of new polymer hydrolysing enzymes. Moreover, these results indicate that arylesterases could be an interesting enzyme class for identifications of polyesterases.

**Electronic supplementary material:**

The online version of this article (doi:10.1007/s00253-016-7992-8) contains supplementary material, which is available to authorized users.

## Introduction

Biodegradable polyesters were introduced to the markets in the 1980s due to the increasing amounts of polyesters ending up as waste in garbage dumps and the fact that these very stable compounds hydrolyse very slowly. The advantage of this new group of polyesters over the traditional ones was that they were degradable by microorganisms at significant rates.

Poly(1,4-butylene adipate-co-terephthalate) (PBAT) was designed as a co-aliphatic-aromatic polyester that maintained some of the beneficial properties of aromatic polyesters while being easier to biodegrade due to its aliphatic components. Microbial biodegradation of PBAT (Kijchavengkul et al. 2010; Nakajima-Kambe et al. 2009; Trinh Tan et al. 2008; Witt et al. 2001) and other aromatic-aliphatic co-polyesters like poly(ethylene terephthalate)/poly(lactic acid) (Hu et al. 2008; Novotny et al. 2015) has been shown with organisms including species of *Pseudomonas* (Cerda-Cuellar et al. 2004; Novotny et al. 2015). However, few of the enzymes with the ability to hydrolyse these aromatic-aliphatic co-polyesters were identified or characterised. Furthermore, many of these studies only reported loss of polymer mass and did not investigate which ester bonds, aromatic or aliphatic, were actually cleaved.

The serine hydrolase TfH from *Thermobifida fusca* (Kleeberg et al. 2005; Muller et al. 2005), the lipase PfL1 from *Pelosinus fermentans* (Biundo et al. 2015), the esterases Cbotu_EstA and Cbotu_EstB from *Clostridium botulinum* (Perz et al. 2015) and the cutinases HiC from *Humicola insolens,* Thc_Cut1 from *Thermobifida cellulosilytica* (Perz et al. 2016b) and Est1 and Est119 from *Thermobifida alba* (Thumarat et al. 2015; Thumarat et al. 2012) have all been reported to be able to hydrolyse PBAT. The producers of these enzymes are typical representatives of compost microbial populations or anaerobic environments (*Clostridium*). There is considerable less known about enzymes from typical aquatic microorganisms related to polyester degrading enzymes. Hence, *Pseudomonas pseudoalcaligenes*, as a typical aquatic microorganism, whose relatives have been reported to be involved in hydrolysis of other aromatic-aliphatic co-polymers (Novotny et al. 2015) and their building blocks (Cerda-Cuellar et al. 2004), was chosen for a proteomic-based screening towards polyesterases. All PBAT degrading enzymes previously investigated were identified either through functional screenings followed by chromatographic purification of the enzyme with subsequent peptide analysis for protein identification, or through in silico screening of publically available databases followed by cloning and expression in a host organism. However, the potential of proteomic screenings of the secretome of microorganisms to identify new PBAT degrading enzymes has not yet been exploited. There are several reasons why this approach would be able to contribute to the discovery of new polyester hydrolases.

Polyesters like PBAT are insoluble polymers that thus cannot be directly taken up by microorganisms. This means that for the polymer to be hydrolysed and potentially utilised, the polymer has to be hydrolysed outside the organism, which in turn leads to the presumption that the degradation is done by a secreted enzyme. By exploiting this idea, we could reduce the complexity of the sample to be analysed by restricting the search to a microorganism’s secretome rather than its entire proteome.

Additionally, identification of an enzyme at the protein level rather than at the genomic level can remove problems related to non-functional genes that might arise due to incorrect gene-splicing recognition or different codon usage by different organisms (Behrens et al. 2011) which in turn could lead to non-functional enzymes. Also, proteomics has the major advantage that, like functional screening followed by protein isolation and identification, truly novel enzymes for a specific function can be identified even though their sequences, structures or regulation are very dissimilar to already reported proteins with the desired function (Sturmberger et al. 2015).

In this work, we demonstrate how a proteomic screening of the secretome of *P. pseudoalcaligenes* led to the identification of an esterase that is induced by the presence of PBAT and that, when recombinantly expressed, can hydrolyse the co-aromatic-aliphatic polyester PBAT.

## Experimental procedures

### Chemicals and materials

The PBAT film and aromatic polyester mimicking oligomer bis(4-(benzoyloxybutyl)terephthalate (BABuTABuBA) were synthesised as previously described (Perz et al. 2016a) and kindly provided by BASF SE (Germany). BABuTABuBA was designed to contain bonds between aromatic and aliphatic moieties; the hydrolysis of which is known to be the limiting step in hydrolysis of polyesters (Perz et al. 2016b). The properties of the PBAT film were as follows: thickness of 50 μm, glass transition temperature (Tg) of −34 °C, melting temperature of 125.3 °C, molecular weight of 65,000 g/mol, M_w_/M_n_ of 3.4 and a crystallinity of ~10% (Perz et al. 2016b). Poly(L-lactic acid) PLLA (PLA) film, thickness 0.05 mm (LS373275 M K S) (crystallinity of 60–70%), and amorphous poly(ethylene terephthalate) (PET) film, thickness 0.25 mm (LS406760), were purchased from Goodfellow (UK). *P. pseudoalcaligenes* subsp. *pseudoalcaligenes* (DSM 50188) was obtained from Leibniz-Institute DSMZ-German Collection of Microorganisms and Cell Cultures in freeze-dried form. All solvents used were of HPLC grade. Unless stated otherwise, chemicals were purchased through Sigma-Aldrich.

### Induction of *P. pseudoalcaligenes* and collection of supernatant

*P. pseudoalcaligenes* was cultured in suspension in media consisting of 5 g/L soy bean peptone and 3 g/L meat extract adjusted to pH 7.0. The culture was kept at 28 °C with orbital shaking at 100 rpm in the dark. For supplementation of media with polymer 0.5 g cellulose (in the form of toothpicks), 1 g milled PBAT or 100 mg of the aromatic polyester mimicking oligomer BABuTABuBA was added to 50 mL of media. Organisms were cultured for either 9 h or 3 days before the supernatant was collected. Fifty-millilitre cultures were transferred to 50-mL conical centrifugation tubes and centrifuged at 4 °C and 4000×*g* for 30 min in a swing-bucket rotor. Supernatant was collected and re-centrifuged as above to yield the final supernatant for analysis.

### Sample preparation of proteomic analysis

Two-millilitre supernatant from each culture condition was centrifuged for 1 h at 10,000×*g* and 4 °C. The supernatant was then transferred to an Amicon Ultra 10 kDa cut-off spin filter (Merck, Germany) and spun at 4000×*g* for 1 h at 4 °C to remove soy bean and bovine peptides originating from the media. Then 4 mL 8 M urea was added to the retentate and spun at 4000×*g* for 30 min at 4 °C to wash the protein suspension. Next, 4 mL 100 mM ammonium bicarbonate was added to the retentate and spun at 4000 x *g* for 1 h at 4 °C. Finally, the retentate was transferred to a 1.5-mL Eppendorf tube and the proteins were precipitated with 6 volumes of acetone at −20 °C overnight. Proteins were pelleted at 10,000×*g* and 4 °C, solvent was removed and the pellet was washed once with 100 μL cold acetone. Proteins were reduced and alkylated by dissolving the pellet in 20 μL buffer containing 10 mM tris(2-carboxyethyl)phosphine, 20 mM iodoacetamide, 4 M urea, 2% sodium dodecyl sulphate (SDS), 0.25 M NaCl and incubated for 1 h at 37 °C at 550 rpm in the dark. Five microlitres NuPAGE LDS sample buffer (4×) and 2 μL NuPAGE sample reducing agent (10×) were added to the sample prior to incubation for 5 min at 95 °C. Sample was loaded on a 20 well NuPAGE 4–12% Bis Tris Midi gel (Invitrogen, USA) and run at a constant 9 W/gel to remove small contaminants. Gel was fixed with 10% ethanol, 7% acetic acid in water and stained with Coomassie Brilliant Blue G. The entire lanes were cut out and cut into seven pieces which were destained using repeated incubations in 25% acetonitrile at 37 °C and 550 rpm. Proteins were digested in-gel by 1.5 μg methylated trypsin (modified porcine trypsin, Promega, USA) in 120 μL 50 mM ammonium bicarbonate, 10 mM CaCl_2_ overnight at 37 °C, 550 rpm. Peptides were extracted from the gel pieces and the extract was dried in a vacuum centrifuge. Dried peptides were redissolved in 20 μL 0.1% formic acid just before analysis by HPLC-MS/MS.

### HPLC-MS/MS analysis

Ten microlitres of the sample was injected and separated by nano-HPLC on an Ultimate 3000 system (Dionex) after enrichment on a PepMap100 C18, 5 μm, 300 μm × 5 mm pre-column (Thermo Scientific, USA) on an Acclaim PepMap RSLC-C18, 2 μm, 75 μm × 50 cm nanoviper nano-column (Thermo Scientific, USA) using the following gradient: solvent A: water, 0.1% formic acid; solvent B: acetonitrile, 0.1% formic acid; 0–5 min 5% B; 5–90 min 5–30% B, 90–90.1 min 30–95% B, 90.1–105 min 95% B, 105–105.1 min 95–5% B, 105.1–120 min re-equilibration at 5% B. Flow rate was 250 nL/min. The separated peptides were directly analysed in an OrbiTrap Velos Pro (Thermo Scientific, USA) in positive ion mode by alternating full scan MS (m/z 300–2000, 60,000 resolution at m/z 200) and MS/MS by CID in the iontrap of the 20 most intense peaks with dynamic exclusion enabled.

### Data analysis

The resulting spectra were analysed by searching a custom database. The database contained all protein entries for *Pseudomonas* in the NCBI database (1,805,231 entries downloaded on Jan. 25th, 2015), a publically available list of common contaminants (http://www.thegpm.org/crap/) and a custom protein entry (GI: 012345) for dimethylated trypsin where all lysines were replaced with the custom amino acid J to avoid false positives resulting from autolysis of modified trypsin (Schittmayer et al. 2016). Searches were performed in Mascot 2.2 (Matrix Science, London, UK). The spectra from the seven gel pieces originating from a single supernatant were analysed together resulting in one protein list per supernatant. Detailed settings: Enzyme: trypsin (with the added specificity of J equal to K) (Schittmayer et al. 2016); max. missed cleavage sites: 2; N-terminus: hydrogen; C-terminus: free acid; carbamidomethylation on cysteine as fixed modification; oxidized methionine, acetylation of N-terminus, demonomethylation of J and dedimethylation of J as variable modifications (Schittmayer et al. 2016); maximum precursor charge 3; precursor mass tolerance +/− 10 ppm; product mass tolerance +/− 0.7 Da; acceptance parameters were 1 or more identified distinct peptides after automatic validation; percolator active (decoy search, with a false discovery rate set below 5%). The mass spectrometry proteomics data have been deposited to the ProteomeXchange Consortium (http://proteomecentral.proteomexchange.org) via the PRIDE partner repository (Vizcaino et al. 2013) with the dataset identifier PXD004014.

### General recombinant DNA techniques

All DNA manipulations described in this work were performed by standard methods (Sambrook et al. 1989). Restriction of DNA was performed using endonucleases *Nde*I and *Hin*dIII (New England Biolabs, USA). Dephosphorylation with alkaline phosphatase (Roche, Germany) and ligation with T4 DNA-ligase (Fermentas, Germany) were done in accordance to the manufacturer’s instructions. Plasmid DNA was isolated by Wizard® Plus SV Minipreps DNA Purification Systems (Promega, Germany) and plasmids and DNA fragments were purified with Promega DNA purification kits. Vector pET26b(+) (Novagen, Merck KGaA, Germany) was chosen for expression of the constructed fusion proteins carrying a C-terminal 6xHisTag in *E. coli* BL21-Gold(DE3) (Stratagene, USA).

### Expression and purification of PpEst

The gene coding for an esterase from *P. pseudoalcaligenes* (Genbank accession number WP_003460012) was codon optimised for expression in *E. coli* and commercially synthesised without signal peptide (GeneArt®, Life Technologies, USA). The gene was cloned over the restriction sites *Nde*I and *Hin*dIII into pET26b(+) and transformed into *E. coli* BL21-Gold(DE3). Freshly transformed *E. coli* BL21-Gold(DE3) cells were used to inoculate 50-mL LB medium supplemented with 40 μg/mL kanamycin that was cultivated at 37 °C and 130 rpm overnight. This culture was used to inoculate 400 mL of fresh medium to an OD_600_ = 0.1 and cultivated at 37 °C and 150 rpm until OD_600_ = 0.6. After cooling down to 25 °C, the expression was induced by addition of isopropyl-β-D-thiogalactopyranosid (IPTG) to a final concentration of 0.05 mM. After expression for 20 h at 25 °C and 140 rpm, the cells were harvested by centrifugation (20 min, 10 °C, 3200×*g*). Pellets from 400-mL culture were suspended in 30-mL NiNTA lysis buffer (20 mM NaH_2_PO_4_, 500 mM NaCl, 10 mM imidazole, pH 7.4) and sonicated with three-times 30-s pulses under ice cooling (Vibra Cell, Sonics Materials, Meryin/Satigny, Swiss Confederation). Lysates were cleared by centrifugation (60 min, 4 °C, 3200×*g*). PpEst was purified by HisTrap FF (GE Healthcare, Germany). Buffer was exchanged for 100 mM Tris-HCl pH 7.0 and purified enzyme stored at −70 °C.

### DNA sequencing, alignments and deposition of sequence data

DNA was sequenced as custom service by Agowa (Germany). DNA analysis was performed with Vector NTI Suite 10 (Invitrogen, USA). BLAST search was performed using the ExPASy (Gasteiger et al. 2003) proteomics server of the Swiss Institute of Bioinformatics, and sequences of related proteins were aligned using the Clustal W program (Swiss EMBnet node server). The nucleotide sequence of PpEst has been deposited in the GenBank database under accession number KX002004.1.

### Hydrolytic activity of non-stimulated and stimulated supernatants

Two hundred microlitres of supernatant were added to 800 µL of 0.1 mM 4-nitrophenyl butyrate (4-NPB) solution pH 8.0 in a 1-mL half-micro cuvette and vortexed briefly before 4-NPB hydrolysis was measured continuously for 10 min by UV-detection of free 4-nitrophenolate at 405 nm on an Agilent Cary 100 UV-Vis spectrophotometer (USA)**.** Autohydrolysis of 4-NPB was determined by adding 0.2-mL freshly prepared medium instead of supernatant.

### Preferred carbon chain length of 4-nitrophenyl carboxylic acid ester substrates

One-millilitre solutions of 0.1 mM 4-nitrophenyl carboxylic esters (with carbon chain lengths from 2 to 14) in 150 mM NaCl, 0.01% Triton X-100 and 20 mM Tris-HCl pH 8.0 were incubated with 1 μg PpEst (in 3.75 μL 100 mM Tris-HCl pH 7.0) at 30 °C for 30 min while the production of 4-nitrophenolate was continuously measured at 405 nm on a Spectramax Omega (BMG-LABTECH, Germany). Autohydrolysis of 4-NPB was determined by the addition of 3.75 μL 100 mM Tris-HCl pH 7.0 without enzyme.

### Temperature optimum of enzymatic activity on 4-NPB

Assay buffer of 150 mM NaCl, 0.01% Triton X-100 and 20 mM Tris-HCl pH 8.0 was heated to temperatures between 25 and 90 °C in 5-degree increments. One microgram of PpEst in 7.5 μL 100 mM Tris-HCl was incubated with 1 mL of the buffer solution for 1 min before 4-NPB was added to a final concentration of 0.1 mM. Production of 4-nitrophenolate was measured continuously at 405 nm for 3 min on a Spectramax Plus (BMG-LABTECH, Germany). Autohydrolysis of 4-NPB was determined by the addition of 7.5 μL 100 mM Tris-HCl (pH 7.0) without enzyme.

### pH optimum of enzymatic activity

PpEst was pre-incubated for 1 h in buffers ranging from pH 4 to 11 (pH 4–6: 0.1 M citric acid buffer, pH 7–9: 0.1 M Tris-HCl, pH 10–11: 0.1 M borate buffer). 4-NPB was added to the assay buffer (150 mM NaCl, 0.01% Triton X-100, 20 mM buffer with appropriate pH) to a final concentration of 0.1 mM and the reaction initiated by adding 1 μg enzyme in 7.5 μL to wells in a 96 well plate. Production of 4-nitrophenolate and 4-nitrophenol was measured at 348 nm (their isosbestic point) (Biggs 1954) continuously for 10 min at 30 °C on a Spectramax Plus (BMG-LABTECH, Germany). Autohydrolysis of 4-NPB was determined by addition of 7.5 μL of the corresponding buffer without enzyme.

### Influence of Ca^2+^ concentration on PpEst activity and stability

First, a 65 μM PpEst solution in 100 mM Tris-HCl pH 7.0 was incubated with 10 mM ethylenediaminetetraacetic acid (EDTA) at 4 °C for 72 h to remove all bound Ca^2+^ and an aliquot was kept for subsequent activity measurement. Then, EDTA was removed from the protein solution by a succession of dilutions in 100 mM Tris-HCl pH 7.0 and ultracentrifugation through an Amicon Ultra 3 kDa cut-off spin filter (Merck, Germany) until EDTA concentration was calculated to be below 10 pM. Subsequently, Ca^2+^ was added to aliquots of the protein solution in ratios of mol Ca^2+^:mol PpEst ranging from 0.05 to 20,000, and the enzyme solutions were incubated for 3 h at 25 °C prior to activity measurement.

For activity determination, 0.5 μg of PpEst (after calcium removal and after readdition of different amounts of calcium) was added to 1 mL 0.1 mM 4-NPB assay buffer (150 mM NaCl, 0.01% Triton X-100, 20 mM Tris-HCl pH 8.0) and production of 4-nitrophenolate was measured continuously at 405 nm for 3 min on a Spectramax Plus (BMG-LABTECH, Germany). Autohydrolysis of 4-NPB was determined by the addition of 3.75 μL 100 mM Tris-HCl pH 7.0 without enzyme. To measure the effect of calcium on protein stability, 50 nM, 10 mM or 200 mM Ca^2+^ was added to solutions of 50 nM PpEst in 100 mM Tris-HCl pH 7.0 after all Ca^2+^ had been removed. The solutions were incubated at 50, 65 or 80 °C for 72 h. After 0, 24, 48 and 72 h, remaining 4-NPB hydrolytic activity was measured as described above.

### *K*_m_ and *k*_cat_ determination

In a 96 well plate, 0.1 μg of PpEst was incubated in 100-μL assay buffer (20 mM Tris-HCl pH 8.0, 150 mM NaCl, 0.1% Triton- X100) at 25 °C with 4-NPB concentrations ranging from 19 μM to 80 mM, and the appearance of 4-nitrophenolate was measured continuously at 405 nm for 3 min on a Spectramax Plus (BMG-LABTECH, Germany). *K*_m_ and *k*_cat_ were determined from functions derived using Sigmaplot 13.0 (Systat Software Inc., Germany) regression wizard equation ‘Exponential Rise to maximum; Single, 2 Parameter’.

### Hydrolysis of polyesters

Milled PBAT with particle sizes between 100 and 300 μm and PBAT, PLA and PET films with the size of 10 mm × 5 mm were used for the hydrolysis experiments of polymers. Prior to incubation, the films were washed with 5 g/L Triton X-100, followed by 100 mM sodium carbonate and subsequently H_2_O. Each washing step was performed at 50 °C in a rotary shaker at 100 rpm for 30 min. To 5 μM PpEst in 1 mL 100 mM potassium phosphate buffer pH 7.0 either 10 mg BABuTABuBA, 10 mg milled PBAT or 100 mm^2^ PBAT, PLA or PET film was added. Polymers were incubated in 2-mL Eppendorf tubes at 50, 65 or 80 °C on a rotary shaker at 100 rpm for 72 h. Samples were taken after 0, 24 and 72 h. Experiments were run in triplicates. In parallel, BABuTABuBA, milled PBAT and PBAT, PLA and PET films were incubated in buffer without enzyme to reveal any autohydrolysis.

Before the analysis of the release products of BABuTABuBA, PBAT and PET, the enzyme was precipitated by addition of ice-cold methanol (1:1 volume/volume) and the samples were acidified to pH 3.0 with HCl. The samples were centrifuged for 15 min at 0 °C and 14,000 rpm (Hermle Z300K, MIDSCI, Missouri, US) and the supernatant collected for further analysis.

Before the analysis of the release products of PLA, the enzyme was precipitated by a modified Carrez precipitation (Carrez 1908; Culhaoglu et al. 2011). Samples were acidified to pH 4.0 with HCl, 20 μl of Carrez reagent I was added and samples were mixed and incubated for 1 min at 25 °C. Then, 20 μl Carrez reagent II was added to the mixture which was mixed and incubated for 5 min at 25 °C. The samples were centrifuged for 30 min at 25 °C and 14,000 rpm (Hermle Z300K, MIDSCI, Missouri, US). Finally, supernatants were filtered through 0.45-μm nylon filters.

### Quantification of hydrolysis products

The samples from PBAT and BABuTABuBA hydrolysis were analysed by HPLC-UV on a system consisting of a Dionex UltiMate 181 3000 pump (Dionex Cooperation, USA), a Dionex ASI-100 automated sample injector, a Dionex UltiMate 3000 column compartment and a Dionex UVD 340 U photodiode array detector. The hydrolysis products were separated by a reversed phase column (XTerra® RP18, 3.5 μm, 3.0 mm × 150 mm) with the recommended pre-column (Waters Corporation, USA) using a non-linear gradient: solvent A: H_2_O, solvent B: acetonitrile, solvent C: 0.1% formic acid; solvent C is at 20% constantly; 0–4.5 min 8% B; 4.5–5 min 8–20% B; 5–13 min 20–30% B; 13–17 min 30–50% B; 17–17.5 min 50–80% B; 17.5–18 min 80% B; 18–19 min 80–8% B; 19–25 min 8% B. All eluents were of HPLC grade. The injection volume was 5 μL and the flow rate was 0.5 mL/min. The column compartment was kept at 25 °C. The expected release products terephthalic acid (TA), 4-(4-hydroxybutoxycarbonyl)benzoic acid (BuTA) and benzoic acid (BA) were detected via UV spectroscopy at 241 and 228 nm. The release products were qualified and quantified by external calibration curves. The reported values for release of products have had the values from the control samples with no enzyme subtracted. The values seen in the controls with no enzyme also showed that the acidification by HCl did not lead to hydrolysis of the polymers.

PLA samples were analysed as published (Pellis et al. 2015), while PET samples were analysed by a modified version of the published method (Herrero Acero et al. 2011) described in detail in the supplementary material.

### Inhibition of PpEst by BuTA

One hundred nanograms of PpEst were incubated in 100 μL 100 μM 4-NPB in assay buffer (20 mM Tris-HCl pH 8.0, 150 mM NaCl, 0.1% Triton –X 100) in a 96 well plate together with BuTA in concentrations ranging from 0 to 1 mM. Activity was determined by the appearance of 4-nitrophenolate at 405 nm on a Spectramax Plus (BMG-LABTECH, Germany) and is given as activity relative to rate when no BuTA was present. K_I_ was determined from the function derived using the Sigmaplot 13.0 (Systat Software Inc., Germany) regression wizard equation ‘Ligand binding; one site competition’.

### Sequence comparisons

The amino acid sequence of PpEst was aligned to the sequences of the known PBAT hydrolysing enzymes TfH, Cbotu_EstA, Cbotu_EstB, HiC and Thc_Cut1 individually using blastp with the BLOSUM62 algorithm with default settings. Protein similarity is given as percentages.

### Modelling of PpEst

The model of PpEst was generated using the Phyre2 server (Kelley et al. 2015) applying the crystal structure of TesA from *Pseudomonas aeruginosa* (expressed in *E.coli*) (PDB code: 4jgg, resolution 1.9 Å) as a template as it has 70% sequence similarity to PpEst. The tetrahedral intermediate was built using the Phyre2 model and BABuTABuBA as substrate. For the energy minimization, YASARA structure (15.10.18) was used (Krieger et al. 2002). Methyl acetate modelled as oxyanion was placed into the active site to form the proposed tetrahedral intermediate by pointing the deprotonated oxygen to the oxyanion hole (main chain of Gly47 and ND2 of Asn76). The active site Ser in the model had to be rotated (the oxygen was positioned at the same location as in the template which was used for modelling). The histidine (His160) was treated as protonated (HIP). Hydrogens were added and the covalently bound intermediate was treated as charged (−1). The model was cleaned and hydrogen bonding networks were optimised. The AMBER03 force field (Duan et al. 2003) was used to apply partial charges. This complex was energy minimised using YASARA structure applying the standard energy minimization protocol. The all-atom minimization root-mean-square deviation (RMSD) of the complex after energy minimization was 0.761 Å compared to the Phyre2 model. Additional functional groups of the substrate were then added successively followed by energy minimization steps until the final complex was created.

## Results

### Extracellular esterase activity of *P. pseudoalcaligenes*

Activity of potential extracellular hydrolases of *P. pseudoalcaligenes* was measured using 4-nitrophenyl butyrate as substrate. Although activity was seen (data not shown), there was no induction of hydrolytic activity by milled PBAT when compared to a control without PBAT, and it was observed that the organism was also not able to use PBAT as its sole carbon source. However, addition of milled PBAT still led to a distinct profile of secreted proteins.

To identify the enzymes responsible for the hydrolytic activity, the supernatants were subjected to proteomic screening. Across the four cultures induced or not induced with different poly- and oligomers (cellulose, milled PBAT or BABuTABuBA), a total of 4081 ungrouped proteins were identified. Among these, 25 were predicted to have esterase, lipase, cutinase or depolymerase activity (excluding DNA or RNA depolymerases) (Table 1). These enzyme classes were chosen as they were previously reported to hydrolyse polymers. Several candidates from the list were considered for cloning and expression. As the target enzyme should preferably be able to cleave PBAT, a desired ability was to hydrolyse ester bonds adjacent to aromatic rings as well as aliphatic esters. Thioesterases cleave ester bonds next to sulfurs and were thus excluded. Phosphodiesterases and phospholipases were likewise excluded due to high substrate specificity for phospho(di)esters. Two enzymes (GI: 610708963 and GI: 667953879) containing transmembrane segments were excluded as these segments might negatively affect stability in solution. Due to the incomplete genomic sequence of *P. pseudoalcaligenes*, not all proteins in the sample could be identified with the *P. pseudoalcaligenes* protein database. Thus, the protein sequences of closely related organisms (all *Pseudomonas* proteins in NCBI) were included as they were assumed to be closely related in sequence to *P. pseudoalcaligenes* proteins. This allowed experimental MS spectra to be assigned to these closely related proteins from other organisms instead of being potentially falsely assigned to unrelated *P. pseudoalcaligenes* proteins. However, these protein hits from other species were subsequently excluded from consideration for cloning and expression as the protein sequence of these enzymes might not be exactly the same in *P. pseudoalcaligenes* and thus not reflect the analysed protein. Of the remaining enzymes, an esterase (GI: 610711757) (from now on named PpEst) was chosen for cloning and expression due to being identified only in the supernatant induced by PBAT, containing a signal peptide for secretion, originating from the correct organism and having a 70% sequence similarity to TesA (GI: 530537561) from *P. aeruginosa* which is known to have arylesterase activity (Kovacic et al. 2013).

**Table 1:** List of esterases and lipases identified in one or more of the supernatants of cultures of *P. pseudoalcaligenes* grown with standard media or with the addition of cellulose, PBAT or BABuTABuBA

GI accession#	Protein name	No polymer added	Cellulose	BABuTABuBA	PBAT
667953335	Glycerophosphodiester phosphodiesterase (*Pseudomonas mendocina* S5.2)	X	X	X	X
782988588	ACP phosphodiesterase (*Pseudomonas pseudoalcaligenes*)	X	X	X	X
610712135	Esterase (*Pseudomonas pseudoalcaligenes AD6*)	X	X	X	X
674101979	Acyl-CoA thioesterase (*Pseudomonas* sp. 1–7)		X	X	X
782988138	Glycerophosphodiester phosphodiesterase (*Pseudomonas pseudoalcaligenes*)	X	X	X	X
667951325	Glycerophosphodiester phosphodiesterase (*Pseudomonas mendocina* S5.2)	X		X	X
782987573	4-hydroxybenzoyl-CoA thioesterase (*Pseudomonas pseudoalcaligenes*)	X	X	X	X
782987758	Thioesterase (*Pseudomonas pseudoalcaligenes*)	X	X	X	X
610712849	Phosphodiesterase (*Pseudomonas pseudoalcaligenes* AD6)				X
782987757	Thioesterase (*Pseudomonas pseudoalcaligenes*)		X		X
610706739	Phosphodiesterase (*Pseudomonas pseudoalcaligenes* AD6)				X
68345928	Arylesterase (*Pseudomonas protegens* Pf-5)				X
**610711757**	**Esterase (** ***Pseudomonas pseudoalcaligenes*** **AD6)**				**X**
641464984	Thioesterase superfamily protein (*Pseudomonas* sp. P482)	X	X	X	
782986630	Diguanylate phosphodiesterase (*Pseudomonas pseudoalcaligenes*)	X	X	X	
939992149	Glycerophosphoryl diester phosphodiesterase (*Pseudomonas syringae* pv. *alisalensis*)			X	
564833263	Phosphoesterase (*Pseudomonas aeruginosa* VRFPA07)			X	
610714766	Carboxylesterase (*Pseudomonas pseudoalcaligenes* AD6)		X		
568075804	Thioesterase (*Pseudomonas* sp. M1)		X		
610416967	Chemotaxis-specific methylesterase (*Pseudomonas aeruginosa* PA96)	X			
739188356	Acyl-CoA thioesterase (*Pseudomonas oleovorans*)	X			
610708936	Esterase (*Pseudomonas pseudoalcaligenes* AD6)	X			
757245813	Lysophospholipase L2 (*Pseudomonas fluorescens*)	X		X	
667953879	Lipase (*Pseudomonas mendocina* S5.2)		X		
801164442	Lipase (*Pseudomonas kilonensis*)		X		

The esterase selected for cloning is depicted in bold. If the protein was identified under the given condition, it is indicated by X

### Cloning and expression of PpEst

The gene coding for the esterase PpEst from *P. pseudoalcaligenes* (202 AA) was codon optimised and cloned over restriction sites *Nde*I/*Hin*dIII into pET26b(+) for expression in *E. coli* BL21-Gold(DE3) without N-terminal secretion signal peptide. For rapid purification, the 6xHis Tag was C-terminally fused to the enzyme. Heterologous expression of PpEst at 25 and 28 °C resulted in strong protein bands in the soluble fractions around 20 kDa corresponding well to the calculated mass of 20.3 kDa with higher expression of soluble PpEst at 25 °C than at 28 °C (Supplemental Fig. S1).

### Characterisation of PpEst

**Fig. 1:**
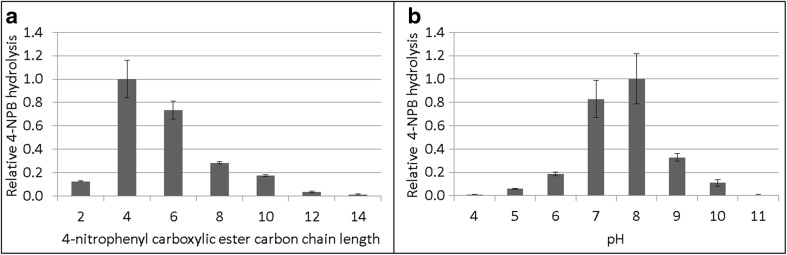
Hydrolysis of 4-nitrophenyl esters and pH optimum. **a** PpEst substrate specificity towards 4-nitrophenyl carboxylic esters with increasing carbon chain lengths. **b** PpEst activity towards 4-NPB in buffers ranging from pH 4 to 11. Values are means of triplicates and standard deviations are shown as *bars*

**Fig. 2:**
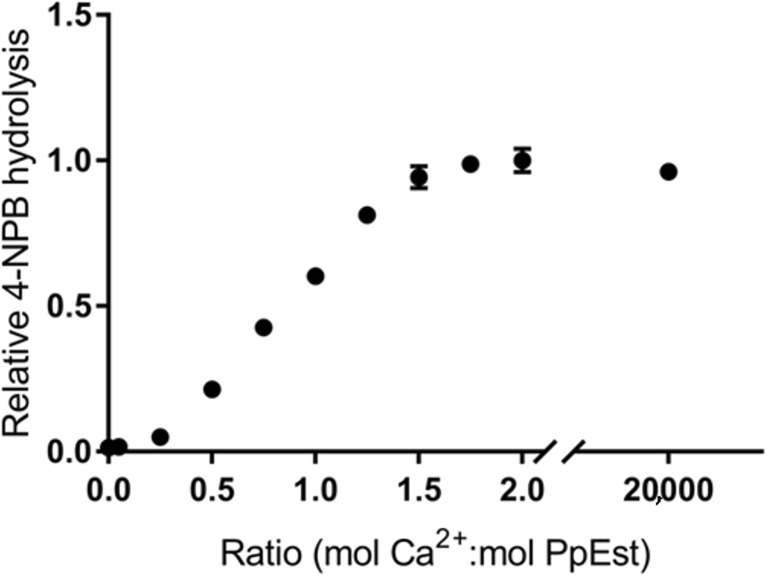
PpEst activity with known Ca^2+^ concentrations. Ca^2+^ was removed from PpEst by complexation to EDTA. Then, EDTA was removed and known amounts of Ca^2+^ were added in ratios of mol Ca^2+^/mol PpEst ranging from 0 to 20,000. Removal of Ca^2+^ completely, but reversibly, abolished PpEst’s activity to hydrolyse 4-NPB. Activity was restored by reintroducing Ca^2+^ to the enzyme, reaching a maximum when the ratio of calcium/enzyme was ~1.25 and did not significantly change with higher calcium concentrations. Values are means of triplicates and standard deviations are shown as *bars*

**Fig. 3:**
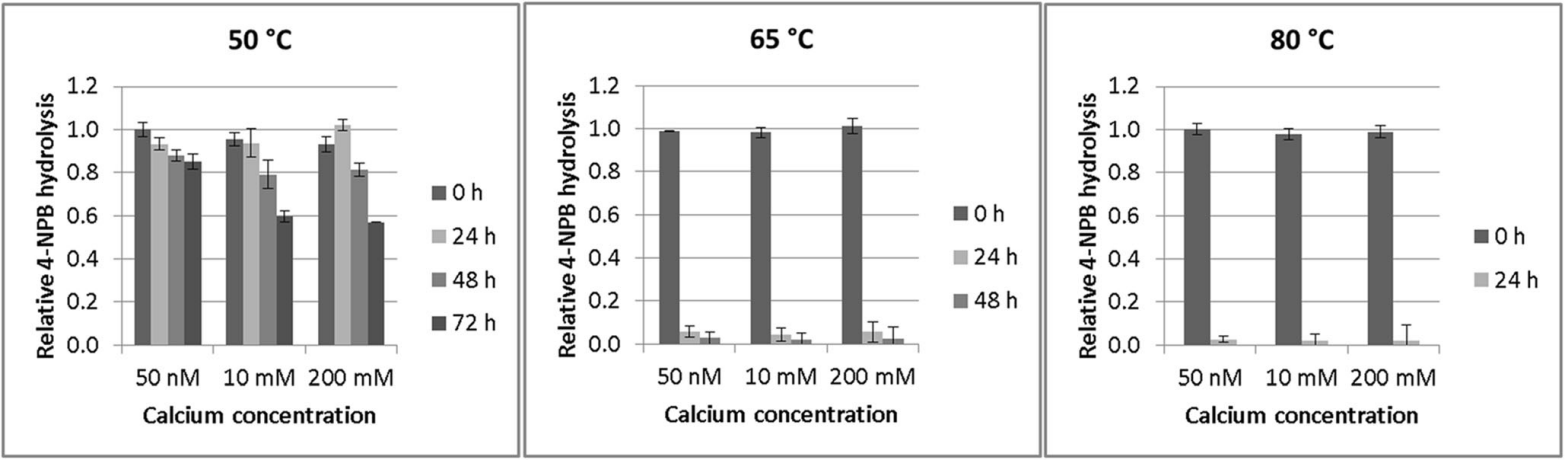
PpEst stability at high temperatures and different Ca^2+^ concentration. PpEst was incubated for 72 h at either 50, 65 or 80 °C with Ca^2+^ concentrations of 50 nM, 10 mM or 200 mM. Remaining 4-NPB hydrolytic activity of PpEst was measured at 25 °C every 24 h either until all activity was gone or 72 h had passed. When Ca^2+^ concentration was 50 nM, 85% of the initial activity remained after 72 h at 50 °C while only 5% activity remained after 24 h at 65 °C and was completely gone after 48 h. At 80 °C, all hydrolytic activity was abolished after 24 h. Higher Ca^2+^ concentrations did not increase PpEst stability. Values are means of triplicates and standard deviations are shown as *bars*

After expression and purification of PpEst from *E.coli* BL21-Gold(DE3), its substrate specificity, pH optimum, temperature optimum and stability and the effects of calcium on activity and stability were determined. Among 4-nitrophenol esters of increasing carbon chain length (C2 to C14), the highest activity was seen towards 4-nitrophenyl butyrate and hexanoate (Fig. 1a). The pH optimum for 4-NPB hydrolysis was found to be between 7 and 8 (Fig. 1b). *k*_cat_ was determined to be 1.98 s^−1^, while *K*_m_ was 4.85 mM for 4-NPB. These values are in the lower and higher ranges, respectively, of values previously reported (Table 2), but, as previously reported, there is not necessarily a direct connection between an enzyme’s ability to hydrolyse the soluble 4-NPB and its ability to hydrolyse insoluble polymers (Ribitsch et al. 2015). PpEst activity towards 4-NPB was not significantly changed between 25 and 80 °C (Supplemental Fig. S2). As other serine hydrolases were found to be stabilised by Ca^2+^ (Bisogno et al. 2003; Diez et al. 1992; Ohto et al. 2005), this effect was also investigated for PpEst. Removing all Ca^2+^ ions by the addition of EDTA resulted in a complete loss of hydrolytic activity. When Ca^2+^ was reintroduced, PpEst activity returned and maximum activity was reached at ~1.25 mol Ca^2+^ per mol PpEst (Fig. 2). Increasing this ratio further did not increase the activity. To test PpEst stability at high temperatures and the effect of increased Ca^2+^ concentrations, 50 nM PpEst solutions were incubated over 3 days at 50, 65 or 80 °C with either 50 nM, 10 mM or 200 mM Ca^2+^. At 50 °C, 85% of enzyme activity remained after 3 days, while at 65 °C remaining activity was only 5% after 1 day and finally at 80 °C no activity remained after 1 day (Fig. 3). Increasing the Ca^2+^ above 50 nM (mol Ca^2+^:mol PpEst = 1) did not increase the stability of PpEst at any of the measured temperatures.

**Table 2:** Comparison of some known PBAT degraders’ *K*_m_ and *k*_cat_ towards the soluble substrate 4-NPB

Enzyme	*k*_cat_ (s^−1^)	*K*_m_ (mM)	Reference
PpEst	1.98	4.85	
TfH	220	0.62	(Zhang et al. 2010)
Plf1	5	6.57	(Biundo et al. 2015)
Cbotu_EstA	71.86	1.95	(Perz et al. 2015)
Cbotu_EstB	5.84	1.30	(Perz et al. 2015)
Thc_Cut1	325	0.8	(Ribitsch et al. 2015)

### PpEst is a polyesterase that can hydrolyse PBAT and BABuTABuBA

**Fig. 4:**
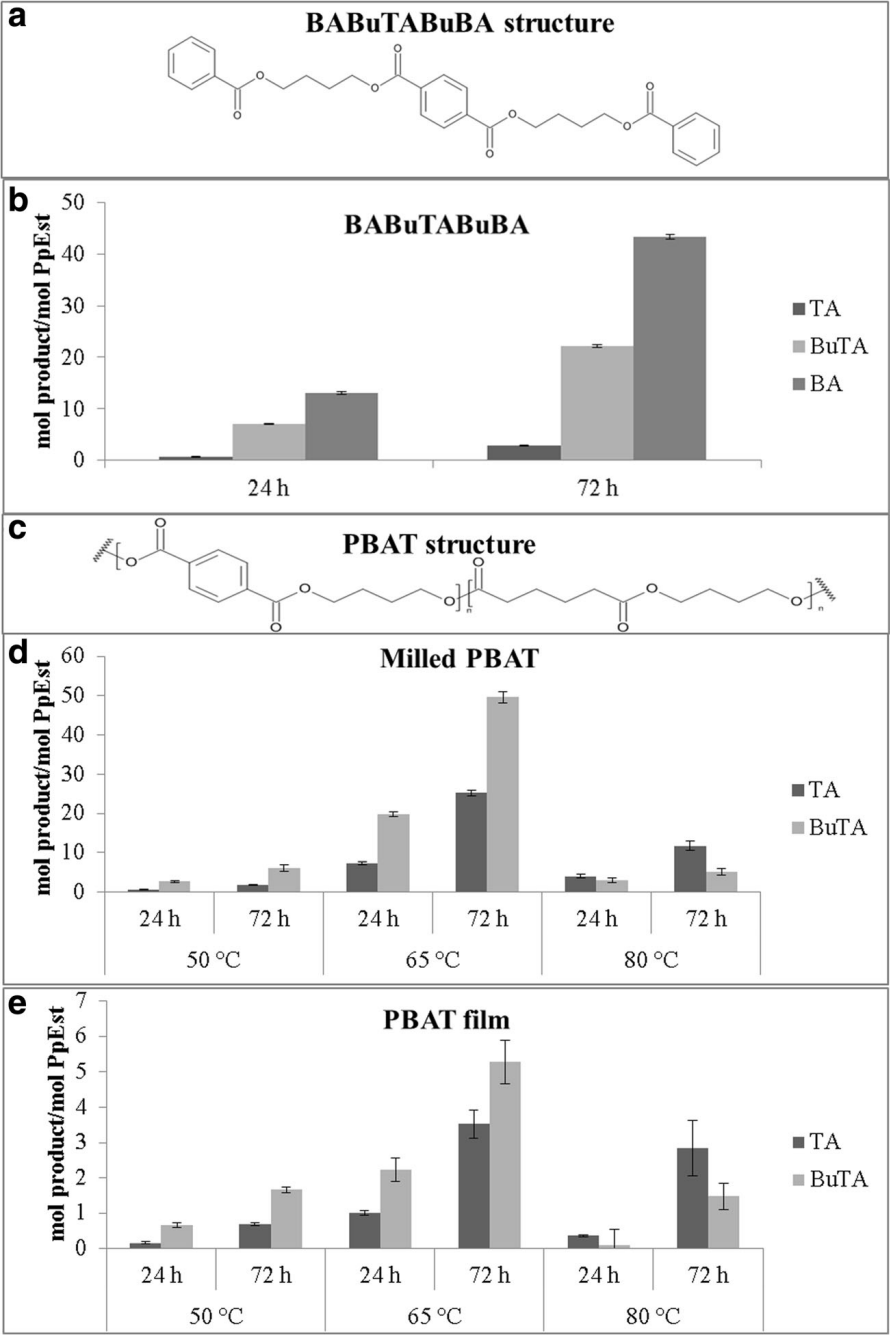
Hydrolysis of BABuTABuBA and PBAT. **a** Structure of the aromatic polyester mimicking oligomer BABuTABuBA. **b** PpEst hydrolysis of BABuTABuTA into its components TA, BuTA and BA at 50 °C after 24 and 72 h. **c** Structure of the aromatic-aliphatic co-polyester PBAT. **d** PpEst hydrolysis of milled PBAT film into its components TA and BuTA at 50, 65 or 80 °C after 24 and 72 h. **e** PpEst hydrolysis of whole PBAT film into its components TA and BuTA at 50, 65 or 80 °C after 24 and 72 h. Values are means of triplicates and standard deviations are shown as *bars*

As PpEst was active on 4-NPB, its activity was tested on the aromatic polyester mimicking oligomer BABuTABuBA (Fig. 4a for structure) to determine if PpEst was able to cleave ester bonds next to aromatic rings. After 72 h at 50 °C, PpEst had released 22 mol TA per mol PpEst (Fig. 4b) demonstrating its esterase activity on an aromatic compound.

Next, we investigated whether PpEst could degrade PBAT (Fig. 4c for structure). First milled PBAT film which is more easily accessible than whole film was tested due to its higher surface area and because milling of the film might have reduced the crystallinity. Hydrolysis of milled PBAT was observed at 50, 65 and 80 °C with the highest activity seen at 65 °C (Fig. 4d).

Moreover, PpEst was also able to hydrolyse whole PBAT film at 50, 65 and 80 °C, again with the highest activity seen at 65 °C (Fig. 4e). The activity at 65 °C was about 7 times higher on the milled film than on the whole film.

Even though only 5% of initial activity remained after 24 h at 65 °C when the enzyme was incubated in a buffer alone, PpEst clearly had remaining activity after 24 h at 65 °C when the polymer was present. This could perhaps be explained by increased protein stability due to adsorbance to the polymer.

To test if PpEst is active also on other polyesters than PBAT, it was incubated with the aromatic polyester PET and the aliphatic polyester PLA. However, no hydrolysis of either polyester could be observed after 7 days of incubation (data not shown).

### PpEst is inhibited by the PBAT hydrolysis product BuTA

The ability of PpEst to hydrolyse BuTA, one of the released degradation products, was tested, but no further hydrolysis could be observed (data not shown). Thus, we hypothesised that the enzyme might be inhibited by BuTA. Indeed, BuTA does inhibit PpEst almost completely (10% activity remaining) at a concentration of 1 mM. *K*_I_ was determined to be 0.67 mM (Supplemental Fig. S3).

### PpEst has low sequence similarity to known PBAT hydrolases

To determine if PpEst was closely related to any known PBAT hydrolases, its sequence was compared to the amino acid sequence of six known enzymes with this function (Table 3). The sequence similarity is lower than 32% for all of them, indicating that PpEst is not closely related to any of the previously known PBAT hydrolases.

**Table 3:** Sequence similarity of PpEst from *P. pseudoalcaligenes* aligned to PBAT hydrolysing enzymes

Enzyme	Similarity to PpEst (%)
TfH	32
Plf1	18
Cbotu_EstA	24
Cbotu_EstB	16
HiC	32
Thc_Cut1	32

### PpEst structure modelled

**Fig. 5:**
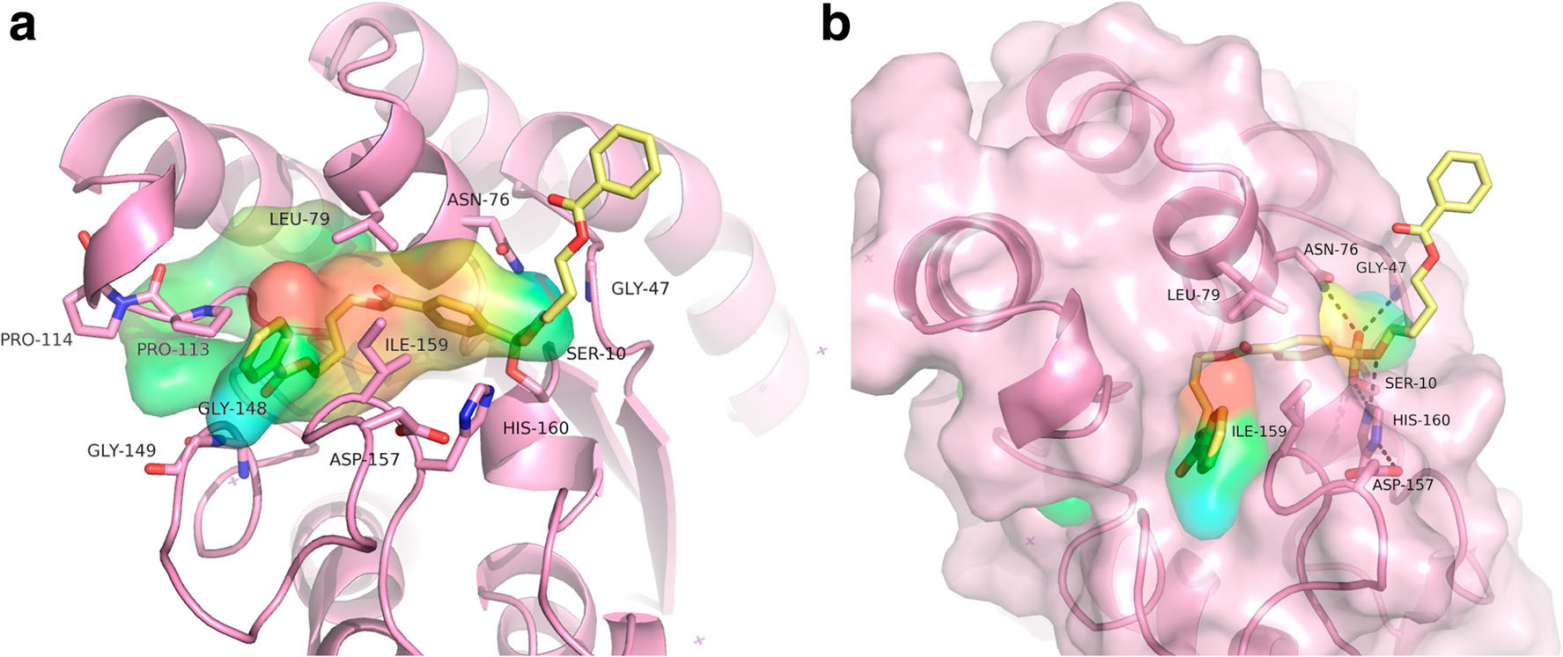
Model of PpEst from *P. pseudoalcaligenes* complexed with BABuTABuBA. **a** Cavity of complex model of PpEst. Leu79, Ile156, Pro113 and Pro114 as well as the loop containing Gly149 and Gly148 may widen the groove on the active site. **b** PpEst model in complex with BABuTABuBA as a tetrahedral intermediate where the active site groove is shown as cavity surface. The predicted catalytic triad consists of Ser10, Asp157 and His160, while the oxyanion hole encompasses Gly47 and Asn76

TesA (PDB code: 4jGG) from *P. aeruginosa* has 70% sequence similarity to PpEst and is thus the enzyme with the highest similarity to PpEst in the RCSB Protein Data Bank. Due to this high similarity and the fact that its structure was determined by X-ray diffraction, it was used as a template to create a model of PpEst to predict the structure of the active site and the surrounding cavity. TesA was described to be a lysophospholipase and to have arylesterase activity (Kovacic et al. 2013) but was not reported to have polyesterase activity. For PBAT degrading enzymes, a sufficient size of the cavity is imperative as it allows access of insoluble polyesters to the active site of the enzyme. When modelling the substrate BABuTABuBA into the active site (Fig. 5a), we observed that the pocket was big enough to accommodate it if minor adjustments were made. The aromatic ring of the substrate did not quite fit into the proposed pocket without moving sidechains. Especially Leu79 and Ile159 were moved slightly during the energy minimization to fit the aromatic ring. This region may also open up a groove through the protein together with a loop (Gly149, Gly148) and a turn on the other side (Pro114, Pro113). This may extend the groove even further relaxing the somewhat restricted substrate geometry in the current model. The cavity surrounding the active site would then be relatively accessible for the solvent indicating that the enzyme could potentially allow large polymers to enter (Fig. 5b). PpEst was found to be dependent on Ca^2+^ to maintain its activity (Fig. 2). However, there is no Ca^2+^ binding domain in this model due to the fact that the template (TesA) does not contain a Ca^2+^ binding site.

## Discussion

Identification of new enzymes for the degradation of polyesters was mainly achieved either by functional screenings followed by protein purification and peptide identification or by in silico searches of publically available genomic databases. Here we demonstrate that secretome screening of microorganisms is an efficient way to identify novel polyesterases from under-investigated environments like aquatic ecosystems with *P. pseudoalcaligenes* being a typical representative.

We studied the extracellular proteome of *P. pseudoalcaligenes* and found that the supernatants of the cultured organism had hydrolytic ability when grown in standard media and when polymers were added to the media. From these supernatants, a total of 25 esterases and lipases were identified (Table 1). Only identified proteins predicted to be secreted enzymes were included for further investigation. Cytosolic proteins may have been identified after cell lysis over the incubation period of 3 days.

Among the identified esterases and lipases, PpEst was found to be induced by the presence of PBAT (as reported with other polyesterases, polymers and organisms (Alisch et al. 2004; Maeda et al. 2005; Wang et al. 2008)). PpEst was predicted to be a secreted enzyme and to have arylesterase activity based on sequence similarity to TesA. To our knowledge, arylesterases have not been reported to act as polyesterases, while, vice versa, several polyester degraders have been shown to be able to use aromatic as well as aliphatic polyesters as substrates. This is the first time, however, that an enzyme was investigated for its polyesterase activity due to its predicted arylesterase activity. We thus suggest that future searches for polyesterases should also include arylesterases in their scope as these enzymes may harbour the desired activities.

PpEst was successfully cloned and expressed in *E.coli*. Its hydrolytic abilities were characterised and the enzyme was modelled. One of the characteristics tested was the role of Ca^2+^ in PpEst activity and stability. Ca^2+^ was necessary for PpEst activity (Fig. 2) and it was observed that ~1.25 mol Ca^2+^ per mole enzyme was necessary for full activity. The influence of Ca^2+^ on PpEst stability at higher temperatures was tested and increasing the Ca^2+^/PpEst ratio above 1.25 did not increase the stability of the enzyme at 50, 65 or 80 °C (Fig. 3). It has to be noted, however, that our model lacks a binding site for this ion (Fig. 5) as the template structure of TesA does not contain a binding site. The model indicates that the active site pocket is relatively open and accessible to the solvent (Fig. 5b), which is imperative for the enzyme to be able to accommodate a polymer.

PpEst hydrolysed the aromatic polyester mimicking oligomer (BABuTABuBA) showing that it was capable of degrading aromatic polyester compounds (Fig. 4b). It was in turn also able to degrade milled PBAT film (Fig. 4d), a polyester substrate that is harder to access due to its size, polymer chain lengths, hydrophobicity and insolubility. Finally, PpEst was tested on whole PBAT film and was able to hydrolyse the polyester even in this inaccessible form (Fig. 4e). This proof of polyester degrading activity demonstrates that proteomic screenings of an organism’s extracellular proteome can lead to the identification of novel polyesterases.

Even though PpEst was able to degrade the aromatic-aliphatic co-polyester PBAT, it was not able to degrade the commercially important aromatic polyester PET nor the aliphatic polyester PLA. PLA had a much higher crystallinity (60–70%) than the PBAT film (10%), which might explain why PpEst did not get access to the polymer chains and thus was unable to hydrolyse them. It is also possible that the active site of PpEst prefers substrates with an aromatic ring coupled to an aliphatic chain of the same length as in PBAT rather than to shorter aliphatic chains (PET), or purely aliphatic polyesters (PLA).

The stability of PpEst at higher temperatures was tested towards 4-NPB after incubation in buffer over 72 h. Only 5% of the initial activity remained after 24 h at 65 °C (Fig. 3). However, when incubated with milled PBAT and PBAT film, PpEst displayed the highest hydrolytic activity at 65 °C (Fig. 4), and the increasing amount of release products after 24 h indicates that PpEst still remained highly active after this time point. This is in contrast to the data on enzyme stability over time in pure buffer. The increased stability in presence of polymers could be explained by adsorbance of PpEst to the polymer. Also, successful polymer hydrolysis is not only dependant on enzyme stability but also on e.g., the crystallinity, melting temperature and glass transition temperature of the polymer (Gan et al. 2004; Perz et al. 2016b). Therefore, it is important to find a compromise between high incubation temperature and enzyme stability as low enzyme stability coupled with high polymer chain mobility (high temperature) might be more effective than high enzyme stability coupled with low polymer chain mobility (low temperature). The increased activity at 65 °C compared to 50 °C could be due to the incubation temperature being closer to the melting temperature of the polymer, resulting in a higher polymer chain motility and thus increased accessibility to the polymeric chains for the enzyme. At 80 °C the hydrolytic activity is lower than at 65 °C suggesting that PpEst is destabilised at this temperature and thus not able to take advantage of the increased polymer chain motility.

The ability of PpEst to degrade PBAT makes it a potential enzyme for use in e.g., waste management plants where polyesters need to be removed or in recycling schemes where the monomers of the polymers are to be recovered for reuse. However, it was observed that the degradation product BuTA (that results from enzymatic degradation of PBAT) inhibits PpEst. In our incubations of PpEst with PBAT films, we only reached concentrations of BuTA that could lead to 10% inhibition, but this could limit the potential PpEst has for PBAT degradation on a large scale. This could however be overcome by removing BuTA as soon as it is produced. One way to do this would be by a cascade reaction done by co-incubation with another enzyme that can hydrolyse this dimer as demonstrated in the case of *Ideonella sakaiensis* 201-F6 where PETase and MHETase collaborate to degrade PET, an aromatic polyester similar to PBAT (Yoshida et al. 2016). This kind of cascade could then also involve using organisms that use terephthalic acid as a carbon source (Zhang et al. 2013) turning this potentially toxic degradation product into biomass that can be reused.

## Electronic supplementary material

ESM 1 (PDF 247 kb)
