# Supplementary material for: PpEst is a novel PBAT degrading polyesterase identified by proteomic screening of Pseudomonas pseudoalcaligenes
Source: Appl Microbiol Biotechnol. 2016 Nov 21;101(6):2291–303. doi: 10.1007/s00253-016-7992-8 (PMC5320007; doi:10.1007/s00253-016-7992-8)
Supplement: Supplementary file 1 — (PDF 247 kb) [file 253_2016_7992_MOESM1_ESM.pdf]

PpEst is a novel PBAT degrading polyesterase identified by proteomic screening of *Pseudomonas pseudoalcaligenes*

Journal: Applied Microbiology and Biotechnology

Paal W. Wallace[a,b,d], Karolina Haernvall[c], Doris Ribitsch[c,f], Sabine Zitzenbacher[b], Matthias Schittmayer[a,d], Georg Steinkellner[b], Karl Gruber [b,e], Georg M. Guebitz[c,f], Ruth Birner-Gruenberger[a,b,d]

a- Research Unit for Functional Proteomics and Metabolic Pathways, Institute of Pathology, Medical University of Graz, Stiftingtalstrasse 24, 8010, Graz, Austria
b- Austrian Centre of Industrial Biotechnology, Petersgasse 14, 8010, Graz, Austria
c- Austrian Centre of Industrial Biotechnology, Konrad Lorenz Strasse 20,3430 Tulln, Austria
d- Omics Center Graz, BioTechMed-Graz, Graz, Austria
e- Institute of Molecular Biosciences, University of Graz, Humboldtstraße 50/III, 8010 Graz, Austria
f- Institute of Environmental Biotechnology, University of Natural Resources and Life Sciences, Vienna, Konrad Lorenz Strasse 20, 3430 Tulln, Austria

Contact info for corresponding author:

Ruth Birner-Gruenberger

Address: Stiftingtalstrasse 24, A-8010 Graz, Austria

Phone number: +43 (0)316 385-72962

Fax number: +43 (0)316 385-73009

E-mail address: ruth.birner-gruenberger@medunigraz.at

Supplementary Methods and materials

Quantification of PLA hydrolysis products

PLA samples were analysed as described by Pellis et al. (Pellis et al. 2015). LC-RI analysis was carried out on a LC system consisting of an HP/Agilent 1100 Series Iso Pump G1310A, an HP/Agilent 1100 Series ALS G1313A automated sample injector, and a HP/Agilent 1100 Series G1316A thermostatic column compartment and refractive index (RI) detector RID G1382A from HP/Agilent LC-RI (Agilent Technologies, Santa Clara, USA). Separation of released hydrolysis products were performed over an ICSep ION-300 column (7.8 mm×300 mm, 7 µm) (Transgenomic, Inc., Omaha, USA). 40 µl of the sample was injected and eluted by 0.01 N H_2SO_4 at a flow rate of 0.325 ml/min at 45 °C. The expected release product lactic acid was detected via RI and qualified and quantified by external calibration curve.

Quantification of PET hydrolysis products

PET samples were analysed by a modified version of the method described by Herrero Acero et al. (Herrero Acero et al. 2011). HPLC-UV analysis was carried out on a HPLC system consisting of Dionex UltiMate 181 3000 Pump (Dionex Cooperation, USA), a Dionex ASI-100 automated sample injector, a Dionex UltiMate 3000 column compartment and a Dionex UVD 340 U photodiode array detector. Separation of released hydrolysis products was performed over a reversed phase column XTerra® RP18 (3.0x150 mm, 3.5 µm) (Waters Corporation, USA). 10 µl of the sample was injected and eluted by an isocratic method consisting of acetonitrile: 0.01 % formic acid: water (1:1:3 (per volume)) at a flow rate of 0.4 ml/min at 25 °C. The expected release products terephthalic acid (TA), mono-(2-hydroxyethyl) terephthalate (MHET) and bis(2-hydroxyethyl) terephthalate (BHET) were detected by UV spectroscopy. Release molecules were qualified and quantified by external calibration curves, where the calibration curve for TA was used to quantify the amount of released MHET as previously described (Vertommen et al. 2005).

Supplementary data

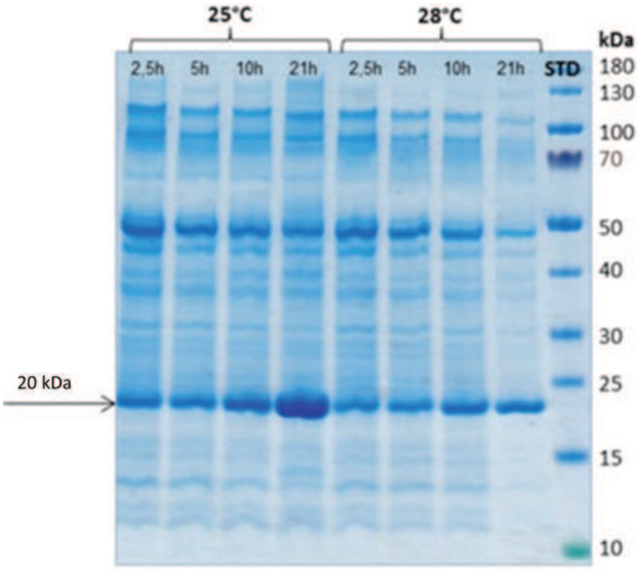

Fig. S1 SDS-PAGE (4-12 %) showing expression of soluble PpEst in *E.coli* BL21-Gold(DE3) at 25 °C and 28 °C at the indicated time points after induction. STD: Standard, PageRuler® Prestained Protein Ladder.

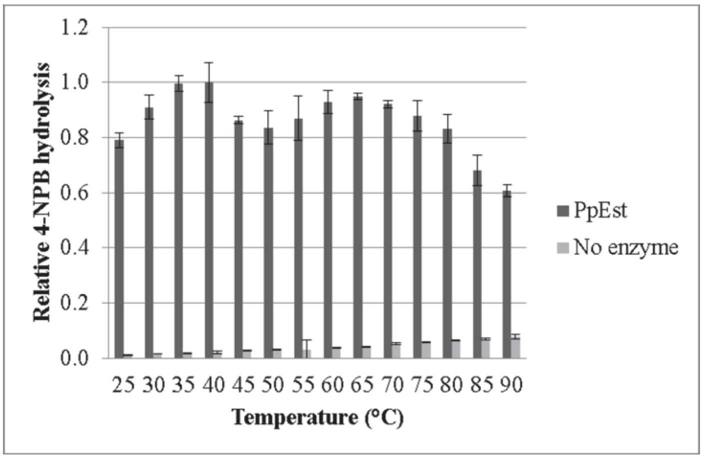

Fig. S2 Hydrolysis of 4-NPB under temperatures ranging from 25°C to 90°C by PpEst. No significant difference in PpEst activity was seen in the range from 25°C to 80°C, but temperatures higher than this decreased the activity. Autohydrolysis (no enzyme) increased slightly with temperature. Values are means of triplicates and standard deviation is shown as a bar.

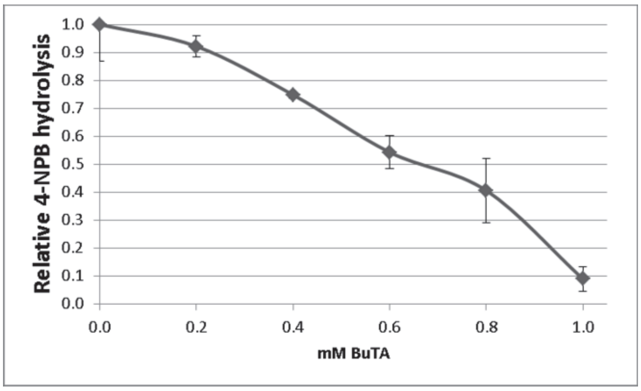

Fig. S3 Relative PpEst hydrolysis of 4-NPB under increasing BuTA concentrations. BuTA inhibits the activity of PpEst resulting in a 90% reduction of activity at 1mM BuTA. Values are means of triplicates and standard deviation is shown as a bar.

References:

Herrero Acero E, Ribitsch D, Steinkellner G, Gruber K, Greimel K, Eiteljoerg I, Trotscha E, Wei R, Zimmermann W, Zinn M, Cavaco-Paulo A, Freddi G, Schwab H, Guebitz G (2011) Enzymatic Surface Hydrolysis of PET: Effect of Structural Diversity on Kinetic Properties of Cutinases from *Thermobifida*. Macromolecules 44(12):4632-4640 doi:10.1021/ma200949p; RP:

Pellis A, Acero EH, Weber H, Obersriebnig M, Breinbauer R, Srebotnik E, Guebitz GM (2015) Biocatalyzed approach for the surface functionalization of poly(L-lactic acid) films using hydrolytic enzymes. Biotechnology journal 10(11):1739-1749 doi:10.1002/biot.201500074

Vertommen MAME, Nierstrasz VA, Veer Mvd, Warmoeskerken MMCG (2005) Enzymatic surface modification of poly(ethylene terephthalate). Journal of Biotechnology 120(4):376-386 doi:10.1016/j.jbiotec.2005.06.015
